# Magnetic keys unlock multichamber capsule robots for smart interventions in the digestive tract

**DOI:** 10.1093/nsr/nwaf486

**Published:** 2025-11-06

**Authors:** Pietro Valdastri

**Affiliations:** School of Electronic and Electrical Engineering, University of Leeds, UK

The incidence of gastrointestinal diseases is increasing worldwide, and their diagnosis and treatment remain challenging [1]. For ingestible capsule devices, most existing technologies can perform only a single task [2,3]. They cannot perform both precise sampling and targeted drug delivery within a one-time procedure [4,5]. The multichamber magnetic capsule robot, developed by the team of Prof. Qingsong Xu and Prof. Yang Lu, provides a novel solution for integrated biopsy and medication in the gastrointestinal tract through a specialized mechanical design and magnetic control.

**Figure 1.**
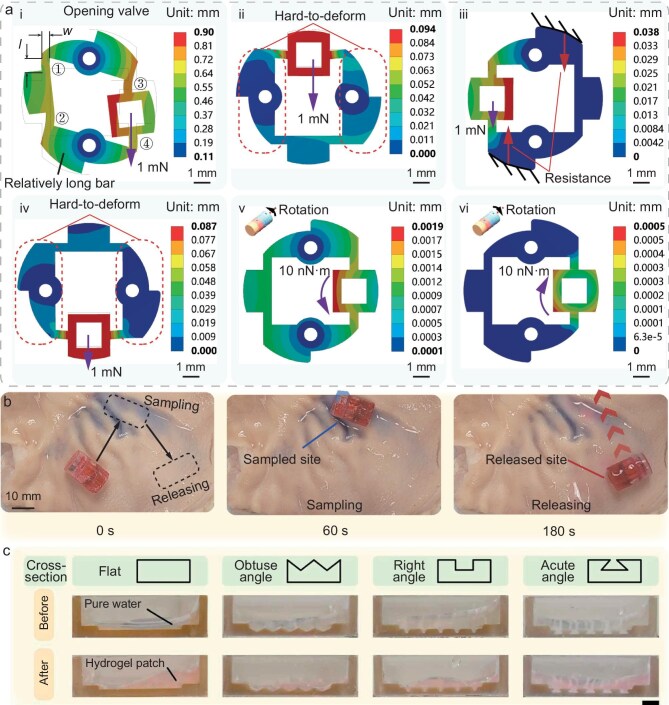
(a) Simulation results of deformation distribution for the magnetic valves under various conditions. (b) Snapshots of the robot for executing liquid sampling and release at different sites. (c) Illustration of *in situ* forming results of the hydrogel patch. Scale bar, 2 mm. Adapted with permission from Ref. [6].

The main contribution of this research is the independent, selective opening of multiple chambers. Each chamber is equipped with a flexible magnetic valve that responds specifically to gradient magnetic fields from certain directions (Fig. 1a). This design exploits the directionality of magnetic force, allowing each chamber to be assigned a unique key and enabling precise control of each chamber. Moreover, this selective opening function is effectively decoupled from the rotating magnetic field that propels the capsule (Fig. 1a), allowing the robot to stably perform liquid sampling or drug release after reaching the target site, thereby avoiding accidental triggering.

At the technical implementation level, the researchers systematically verified the valve’s reliability in opening/closing on demand and its sealing capability through experiments. In the *ex vivo* experiments using a porcine stomach model, the navigation was guided by ultrasound imaging, and the liquid sampling and release at multiple sites demonstrated the clinical potential of this technology (Fig. 1b). It is noteworthy that the researchers further proposed a drug sustained-release strategy based on *in situ* generation of a shape-adopted hydrogel patch (Fig. 1c). By sequentially releasing inclusions from different chambers to form a drug-loaded gel at the target site, this approach enables sustained drug release for treating gastrointestinal diseases.

Another significant contribution of this work lies in its modular design and adaptability. The ability to adjust the number and functions of chambers enables it to meet various clinical requirements, whether for delivering a single drug medication, implementing multidrug combination therapies, sampling bodily fluids from multiple target sites, or performing biopsies and administering medicines within a single procedure. This platform shows great promise as an integrated system for both diagnosis and therapy.

Indeed, several issues must be addressed before translating this technology into clinical practice, such as ensuring motion accuracy within *in vivo* dynamic environments, advancing development at smaller scales, and confirming safety and biocompatibility. Nonetheless, this research clearly advances the development of next-generation intelligent gastrointestinal medical devices, ultimately achieving non-invasive, precise, and personalized diagnosis and therapy.
